# The Prevention of Diarrheal Diseases in Infants

**Published:** 1903-08

**Authors:** Robert W. Hynds

**Affiliations:** Atlanta, Ga.


ATLANTA

Journal-Record of Medicine.

Successor to Atlanta Medical and Surgical Journal, Established 1855, and Southern Medical Record, Established 1870.

VOL. V. AUGUST, 1903. No. 5.

BERNARD WOLFF, M.D., EDITOR, Nos. 319–20 Prudential.

Published Monthly.

M. B. HUTCHINS, M.D., BUSINESS MANAGER, No. 64 Marietta St.

### ORIGINAL COMMUNICATIONS.

### THE PREVENTION OF DIARRHEAL DISEASES IN INFANTS.

BY ROBERT W. HYNDS, M.D.,
ATLANTA, GA.

By far the most frequent cause of gastro-intestinal diseases in infants is food infection, and as only a small percentage of these cases occur in breast-fed infants, an effort should be made to secure a pure food supply. This problem assumes an increased importance when we remember that with our constantly increasing knowledge of the causation of these diseases, there has been no decrease in the mortality.

An increasing proportion of mothers, whether incapacitated by disease or on account of the poor quality or scarcity of their milk, or from a desire to avoid the discomforts of a prolonged lactation, compel us to resort to an artificial food, and time has proven a modified cow's milk to be the best diet. This being so, an effort should be directed toward procuring as pure a product as possible.

The entire subject of the prevention of diarrheal diseases in infants may be crystallized in the one word cleanliness; cleanliness in the dairy, cleanliness in the handling of the milk and cleanliness in the feeding.

How few physicians, especially the general practitioner, investigate the source of the milk they use. Should they do so, they will ofttimes find the cow standing in filth and covered with dirt. They will see great piles of manure banked near a dirty, ill-ventilated barn. They will find musty litter being used, and a sour-smelling milk-room. This is not an unusual picture, and yet physicians sometimes wonder why babies fed on milk from such a dairy suffer from diarrheal diseases.

The majority of intestinal diseases in infants are due directly or indirectly to bacteria. This accounts for the fact that they are more prevalent in the summer, as the bacteria in milk are much more numerous during the hot season. Over two hundred distinct types of bacteria have been found in milk. The bacilli of tuberculosis, typhoid fever and diphtheria have been isolated from milk, and scarlet fever may be produced by milk from cows suffering from that disease. Certain forms of aphthæ, herpes and throat inflammations are caused by milk bacteria, especially the former where the cows are suffering from foot and mouth disease.

But there is a large class of intestinal diseases characterized by diarrhea whose origin cannot be traced to any distinct form. Some of the most violent of these diseases are caused by toxins formed in the milk, while others are due directly to the action of the bacteria introduced into the intestinal canal. Among the distinct forms of bacteria found in milk that produce diarrhea is the bacillus dysenteriæ or bacillus of Shiga. Very violent infections are also due to a type of the streptococcus and some types of the colon bacillus.

The possibility of destroying all the bacteria in milk was heralded with joy, but such a method will probably not be generally used. Heat alters the taste of milk and produces certain chemical changes which impair its digestibility and food value. It modifies the character of the fat emulsion, changes the nature of the proteids, partially converts the sugar into caramel, the soluble lime salts are converted into insoluble ones, and the various ferments and enzymes are destroyed.

While destroying most of the bacteria capable of producing intestinal diseases, heat also destroys the lactic acid forming group which seems to conserve some usual purpose in the intestine, probably by counteracting the action of the putrefactive bacteria normally found in the intestine. It is impossible to pasteurize impure milk and make it wholesome, especially in those cases where poisonous toxins have already been formed.

This being so, our aim should be to obtain a milk free as nearly as possible from those organisms known to produce intestinal disturbances and to feed it raw.

The most common types of bacteria producing diarrhea are those belonging to the peptonizing group.

In one series of two hundred infants fed upon milk obtained under strict sanitary precautions not a single case of diarrhea developed. Laboratory experiments have revealed the fact that the peptonizing bacteria found in milk acting upon albumen give rise to decomposition products which are toxic in their character, and it is to be supposed that like products would be found under like conditions in the intestines.

It is generally supposed that so long as the milk is not sour it is a fit food; this is true only conditionally. A milk may be kept at a low temperature for several days so that the growth of the lactic bacteria will be prevented and the milk will retain its sweetness; but the peptonizing and other forms of bacteria will grow with increased rapidity since the inhibiting power of the lactic acid bacilli is removed, and render the milk unfit for use.

Pure milk at best is rather difficult for an infant to digest on account of the difference in character of the albumen in human and cow's milk. The constituents of milk are specific in character, varying with the species. The young of no species will thrive as readily on the milk of another species as they will on that of their own. At the same time none of the artificial foods have proved as satisfactory as modified cow's milk. This being so, we should study the mode of infection of the milk and the means of preventing it.

*Source of Infection.*—At the time when the milk is secreted it is free from bacteria, so the bacteria commonly present in milk must come from without. It has been demonstrated that an absolutely sterile milk can be obtained from a healthy udder. The udder, however, may be the seat of tuberculosis foci, or may be inflamed as the result of a local or general infection, and in this way may infect the milk. The mastitis is usually caused by some type of the streptococcus, and an abundance of these, together with pus, is found in the milk.

Bacteria is nearly always present in the milk ducts and cistern, gaining entrance to these from an external source through the teats. This contamination usually only takes place in the lower ducts. Most of the common species of milk bacteria will not live within the milk ducts, as for instance the bacillus lactis acidi. The one most commonly found is some type of the streptococcus.

The air is an important mode of milk contamination, especially so if the milking is done in a crowded, ill-ventilated barn, and if a musty bedding is used. A musty dirty straw is a veritable hot-bed for bacteria, and particles of the straw, loaded with bacteria are thrown into the air by the movements of the cow and fall into the milk. Probably the most important source of bacterial contamination is the cow. Much less attention is paid to careful grooming than should be, and the cow is often at the time of milking, covered with dirt and filth ; the movements of her body, or the switching of her tail cause some of these particles to become dislodged and fall into the milk, carrying their load of bacteria, or some of the germ-laden hairs from the cow's flank fall into the pail. The type of the bacteria gaining entrance to the milk from this source is varied, the cocci probably predominating.

If the cow is fed on dry bran or hay during milking, some particles of the feed are almost certain to fall into the milk and contaminate it. The bacteria from the dirty hands or dirty clothes of the milker frequently cause bacteria contamination, especially so if the method of "wet milking" is used, as is very often the custom.

One source from which contamination takes place, and which can scarcely be avoided except at considerable expense, is the milk vessels. If they are not thoroughly washed in hot water each time after using some milk will be left clinging to their sides, especially is this the case if the vessels are battered and worn; if they are rinsed in cold water before use, some of the water is usually left in them and contaminates the milk.

The most dangerous form of contamination is caused by unscrupulous dealers adding water to the milk. This cannot always be detected by chemical tests, and it is to be hoped that cryoscopy will be brought to a practicable working basis within the means of the general practitioner.

The longer the milk is left standing uncovered and the greater the time that elapses before it is cool, the greater will be the number of bacteria.

It is the custom of some of the dairymen of Atlanta to fill the milk jars at the stream which flows from the aerator. The jars often overflow, and this overflow milk is caught in a tub and used after it has passed over the dirty outside of the jar and the hands of the dairyman. It is also the custom in this city to fill the jars as they are collected from the houses of customers without washing them. This practice is positively criminal.

We must remember that all milk contamination cannot be charged against the dairyman, but that careless handling in the home is responsible for some of it.

It has not been definitely decided what number of bacteria renders the milk unfit for infant feeding. Some milk commissions have fixed the maximum number to the cubic centimeter at ten thousand, and others at thirty thousand. This will depend greatly upon the type of bacteria found.

*Reduction of Contamination.*—The stable should be light and well ventilated without a hay loft above. The walls should be kept well whitewashed, the floors should be well drained, and no musty litter should be used. Manure should be removed several times a day and piled not nearer than a hundred feet from the barn. This will tend to reduce the number of flies in the barn. It is better to have a separate milking-room, or if this cannot be done, the cows should be milked in the open air. The cow should be groomed twice a day, and the long hairs clipped from her flanks and tail to prevent them from falling into the milk. No employee should be allowed to come in contact with the cows or handle the milk who has been exposed to or is suffering from any contagious disease. It is important that the cows be well treated, as any fright or ill-treatment produces a change in the milk.

A pure water supply is one of the most important requisites to a sanitary dairy. Impure water from cistern, shallow wells or streams may affect the milk directly or indirectly. Indirectly by injuring the health of the cow, and directly by contamination with bacteria. No well located near a stable or any privy vault but is liable to contain pathogenic bacteria. All milk vessels should be thoroughly washed and then sterilized with boiling water or steam.

Wet milking should never be allowed, as the dirt and bacteria from the hands are washed into the milk. It is a good practice, however, to moisten the cows udder with a damp cloth before milking. As has been before mentioned, streptococci are normally present in the lower milk ducts, but the number of these gaining entrance into the milk may be considerably reduced if the first few streams are thrown upon the ground.

Milk must be removed from the barn as soon as possible, and placed in a clean milk-room. The longer it is left exposed to the air, the greater will be the contamination. The most important point of all in connection with the handling of milk is immediate cooling. This retards the development of bacteria. Milk may be kept at a temperature of fifty degrees for thirty-six hours without souring. The question of aeration is of less importance. If the dairy is clean, the odor attached to the milk will be slight. Before bottling, the milk should be strained through a sterilized cloth, or perhaps better through both a steel gauze and cheese cloth.

Milk that curdles soon after drawing, blue milk, slimy milk and milk becoming bitter some time after drawing are all the result of some form of bacteria, and all such milk should be thrown away. If the cow shows any evidence of mastites her milk should be discarded.

The points mentioned above seem small in themselves, but upon such hang the lives of many a babe. The infantile digestive apparatus is a delicate organism and easily disturbed. We should do all in our power to protect it, for when the equilibrium is once overthrown it is hard to restore. Preventive medicine is the acme of the medical science of this century, and this is one of the phases of the subject we should not neglect. The percentage of deaths from diarrheal diseases in infants in our cities to day is not proportionate to our knowledge of the disease or of our means of preventing it.

By the enactment of proper rules governing dairies and the enforcement of the same by the Boards of Health in our cities the bacterial contamination of milk can be reduced 95 per cent. The dairies should be regularly inspected by competent experts, and the milk submitted to a chemical and bacteriological examination. A certificate can then be issued to the dairy whose general condition and the character of its product warrants it, and such milk can be known as certified milk. This milk will demand a slightly higher price than non-certified milk, but on account of its increased purity will be well worth the extra cost.

